# Identification of Proteins Involved in Carbohydrate Metabolism and Energy Metabolism Pathways and Their Regulation of Cytoplasmic Male Sterility in Wheat

**DOI:** 10.3390/ijms19020324

**Published:** 2018-01-23

**Authors:** Xingxia Geng, Jiali Ye, Xuetong Yang, Sha Li, Lingli Zhang, Xiyue Song

**Affiliations:** College of Agronomy, Northwest A&F University, Yangling 712100, Shaanxi, China; gengxingxia@nwafu.edu.cn (X.G.); yejiali@nwafu.edu.cn (J.Y.); yangxuetong@nwafu.edu.cn (X.Y.); lisha2012@nwafu.edu.cn (S.L.); zhanglingli@nwafu.edu.cn (L.Z.)

**Keywords:** anther proteomics, cytoplasmic male sterility, hybrid wheat, isobaric tags for relative and absolute quantification (iTRAQ)

## Abstract

Cytoplasmic male sterility (CMS) where no functional pollen is produced has important roles in wheat breeding. The anther is a unique organ for male gametogenesis and its abnormal development can cause male sterility. However, the mechanisms and regulatory networks related to plant male sterility are poorly understood. In this study, we conducted comparative analyses using isobaric tags for relative and absolute quantification (iTRAQ) of the pollen proteins in a CMS line and its wheat maintainer. Differentially abundant proteins (DAPs) were analyzed based on Gene Ontology classifications, metabolic pathways and transcriptional regulation networks using Blast2GO. We identified 5570 proteins based on 23,277 peptides, which matched with 73,688 spectra, including proteins in key pathways such as glyceraldehyde-3-phosphate dehydrogenase, pyruvate kinase and 6-phosphofructokinase 1 in the glycolysis pathway, isocitrate dehydrogenase and citrate synthase in the tricarboxylic acid cycle and nicotinamide adenine dinucleotide (NADH)-dehydrogenase and adenosine-triphosphate (ATP) synthases in the oxidative phosphorylation pathway. These proteins may comprise a network that regulates male sterility in wheat. Quantitative real time polymerase chain reaction (qRT-PCR) analysis, ATP assays and total sugar assays validated the iTRAQ results. These DAPs could be associated with abnormal pollen grain formation and male sterility. Our findings provide insights into the molecular mechanism related to male sterility in wheat.

## 1. Introduction

Heterosis has important roles in agricultural production throughout the world, where cytoplasmic male sterility (CMS) is an effective approach that has been investigated in many crops such as Brassica napus [1], rice [2,3] and soybean [4]. CMS is widespread in flowering plant species [5] where it is characterized by the inability of plants to produce functional microspores, pollen or anthers, or male gametes. In CMS plants, the pistil still has the capacity to accept fertile pollen from other normal plants and produce seeds. The CMS system is utilized widely in hybrid crop breeding to avoid the extra effort required for artificial emasculation. 

Wheat (*Triticum aestivum* L.) is the most widely planted and important food crop. Heterosis can be employed to increase the wheat yield and it has been applied widely in hybrid wheat breeding. Previously, the utilization of heterosis in common wheat (*Triticum aestivum* L.) production has mainly been achieved via CMS-based breeding methods, i.e., the three-line system and chemical hybridizing agents (CHAs) [6,7]. However, some constraining factors may delay hybrid wheat production, such as the time required, expense, damaging cytoplasmic effects and the genetically complex fertility restoration processes required for the three-line system [8], as well as phytocidal effects, low seed germination rates and potential chemical residues due to the use of CHAs. Currently, a two-line system involving thermo-sensitive CMS (TCMS) is being studied to assess its possible application in hybrid wheat breeding. This system can maintain and multiply male sterile lines via self-pollination, which is much simpler and cheaper than a three-line system in wheat [9].

Proteomics can be used an effective tool for understanding the functions and interactions of genes and it has been employed previously to detect differences in the expression levels of proteins in the sterile and fertile anthers of several plant species, thereby giving insights into the possible mechanisms related to plant sterility. Proteomics techniques have been used to identify several proteins related to male sterility in wheat, which are involved in protein synthesis, cell death, signal transduction and carbohydrate metabolism [10]. In addition, proteomics analysis demonstrated that proteins associated with carbohydrate and energy metabolism, flavonoid synthesis and photosynthesis may play roles in pollen development because they are downregulated in the anthers of CMS Brassica napus [11]. In addition, differential proteomics studies of the wild type wolfberry and a male sterile mutant showed that the levels of several proteins were lower in the sterile anthers and they may be related to male sterility [12]. These studies yielded novel insights into the molecular mechanism related to CMS.

Recently, the isobaric tags for relative and absolute quantification (iTRAQ) method was developed as a powerful approach to proteomics analysis. This method is very useful for obtaining the protein profiles of tissues and comparing the different protein expression levels in various cells or tissues. iTRAQ-based differential proteomic analysis has been used to study the development of anthers in plants with male sterility, such as cybrid pummelo [13], cotton [14] and soybean [4] but CMS has not been investigated in wheat using this technique.

In this study, we are dedicated to identifying proteomic differences between a male sterile line and a maintainer line by proteomic sequencing techniques with the aim to help understand the mechanism of pollen abortion in wheat and to identify fertility related candidate proteins.

## 2. Results

### 2.1. Phenotypic Identification and Cytological Observations

**Figure 1:**
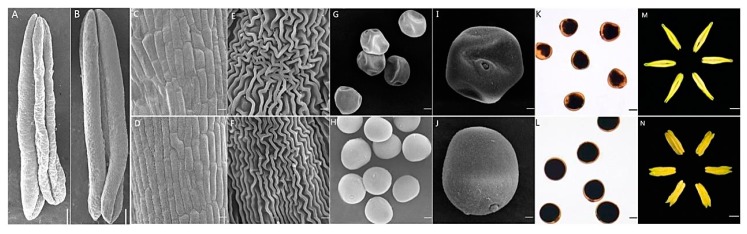
Scanning electron microscopy observations of sterile (**A**,**C**,**E**,**G**,**I**,**K**,**M**) and fertile (**B**,**D**,**F**,**H**,**J**,**L**,**N**) wheat plants during the trinucleate (TNP) stage. (**A**,**B**,**M**,**N**): anthers; (**C**,**D**): outer epidermal cells; (**G**,**H**,**I**,**J**): TNP cells. Bars: (**A**,**B**): 0.5 mm; (**C**,**D**,**G**,**H**): 100 μm; (**E**,**F**): 10 μm; (**I**,**J**): 30 μm; (**K**–**N**): 50 μm.

The wheat CMS line KTM3315A was developed via consecutive backcrosses with TM3315B as the donor parent [9]. KTM3315A, a TCMS line, was developed by Northwest A&F University, China, where its thermo-sensitive fertility depends on the recessive nuclear gene *rfv*_1_^ma^ from the 1BS chromosome of *Triticum macha* [15]. KTM3315A is completely male sterile at temperatures <18 °C during Zadoks growth stages 45 to 52 and it is capable of producing self-pollinated seeds when the temperature exceeds 20 °C in this growth period. In order to observe the phenotypic and cytological differences in the anthers of KTM3315A and its maintainer TM3315B during the wheat growth season, morphological landmarks or cellular events visible in the anthers (or pollen) were observed by light microscopy and scanning electron microscopy (SEM). In contrast to the anthers of TM3315B, the anthers of KTM3315A were thin and shriveled and the upper part did not dehisce to release mature pollen grains in the trinucleate (TNP) stage (Figure 1A,B). The anther epidermal cells were more irregular in shape in KTM3315A compared with TM3315B (Figure 1C–F and Figure S1) and the pollen grains of KTM3315A were also irregular (Figure 1G,I). In order to determine the activity of the pollen grains, anthers from the two lines in the TNP stage were stained with I_2_–KI. In contrast to the fertile mature pollen (Figure 1K,L), the sterile pollen grains could not be stained deeply, thereby indicating that they were not viable.

**Figure 2:**
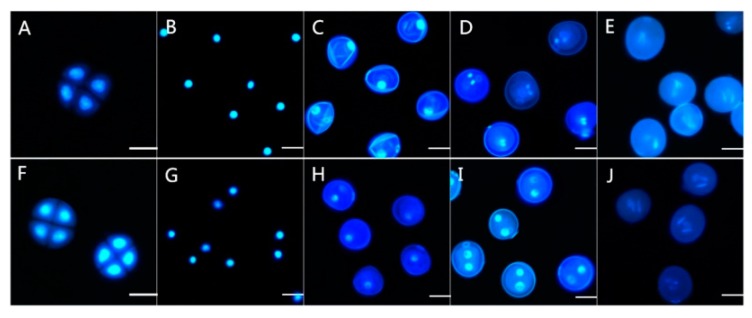
DAPI stained pollen from KTM3315A (**A**–**E**) and its maintainer line TM3315B (**F**–**J**). (**A**,**F**) Tetrad stage; (**B**,**G**) early uninucleate stage; (**C**,**H**) later uninucleate stage; (**D**,**I**) binucleate stage; and (**E**,**J**) trinucleate stage. Bar: 50 μm.

Moreover, we investigated the development of the microspores from sterile plants and fertile plants by 4′,6-diamidino-2-phenylindole (DAPI) staining (Figure 2). Before the early uninucleate (UNP) stage, there were no significant differences between the TM3315B and KTM3315A plants (Figure 2A,B,F,G). However, in the later UNP stage, cell development was obviously abnormal and folds were present in the cells from KTM3315A plants (Figure 2C) but not in those from TM3315B plants (Figure 2H). As the cell and nucleus developed, the nucleus had a smaller and more concentrated shape during the binucleate (BNP) stage in the sterile plants (Figure 2D). In the TNP stage, there were obvious differences between the mature pollen grains, which contained two sperm nuclei and a vegetative nucleus in the fertile plants (Figure 2J), whereas the cell nuclei were malformed in the sterile plants during this stage (Figure 2E). Therefore, abnormalities of the sperm nucleus in the pollen during the TNP stage finally caused male sterility in wheat.

### 2.2. Primary Data Analysis and Protein Identification

In order to clarify the molecular mechanism responsible for CMS at the protein level, we performed proteomics analyses with the iTRAQ method using the anthers obtained from three development stages in the sterile line KTM3315A and its maintainer line TM3315B.

In total, 209,442 spectra were obtained in the iTRAQ experiment using anthers from the sterile line and maintainer line. Based on these spectra, we identified 73,688 known spectra, 33,464 unique spectra, 23,277 peptides, 13,124 unique peptides and 5570 proteins (Figure S2A). The number of proteins decreased as the number of peptides that matched with the proteins increased but >67.4% of these proteins included at least two peptides (Figure S2B). Figure S2C,D show the protein mass distribution and sequence coverage distribution. Thus, proteins with masses of 20–30 kDa and 30–40 kDa had the greatest abundances, followed by proteins with masses of 40–50 kDa and 10–20 kDa. The number of proteins decreased as the sequence coverage increased. Figure S2E shows the distribution of the peptide lengths in wheat anthers, which indicates that <12.2% of all the peptides contained ≥20 amino acid residues and 83.1% comprised 9–20 residues. Detailed information about the proteins identified is given in Tables S1 and S2.

### 2.3. Identification of DAPs by iTRAQ

**Figure 3:**
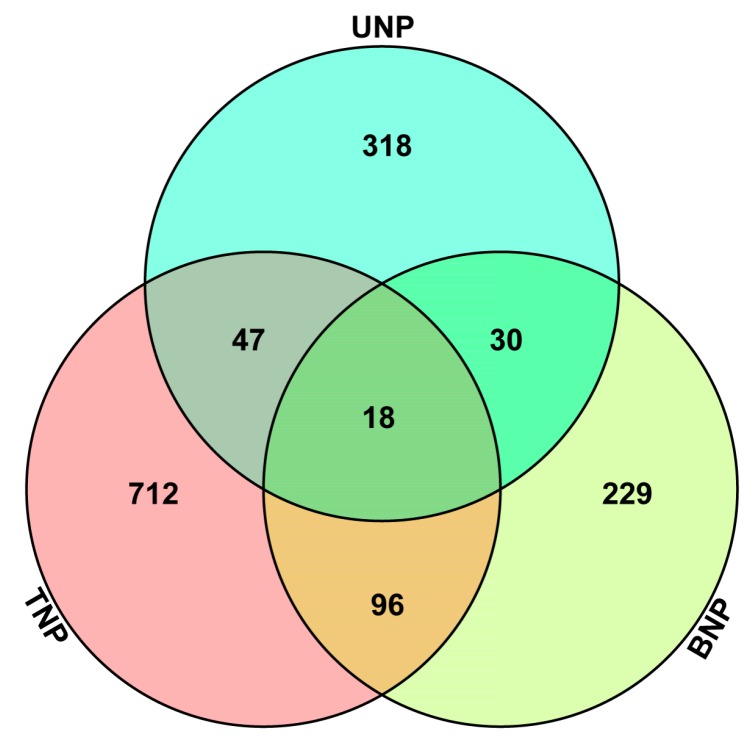
Venn diagrams and expression levels of 1450 differentially abundant proteins (DAPs) identified by iTRAQ in wheat anthers during different development stages. The numbers of DAPs are shown for different developmental stages. UNP, BNP and TNP represent the numbers of DAPs in KTM3315A and TM3315B during the uninucleate stage, binucleate stage and trinucleate stage, respectively.

In total, iTRAQ detected 5570 non-redundant proteins. The thresholds used for determining significant differences in protein expression were “*P* ≤ 0.05 and |FC| ≥ 1.2”. Using these two criteria, we identified 1450 DAPs in KTM3315A and TM3315B and their details are given in Table S3. The distributions of the 1450 DAPs as well as their overlapping in different developmental stages were visualized by Venn diagram analysis, as shown in Figure 3, which indicates that 18 DAPs were expressed in all of the developmental stages, 173 DAPs in two stages (47 in UNP and TNP, 96 in BNP and TNP and 30 in UNP and BNP) and 318, 229 and 712 DAPs only in the UNP, BNP and TNP stages, respectively. Thus, significantly more DAPs were identified during the TNP stage than the other two stages.

### 2.4. Functional Classification of DAPs

**Figure 4:**
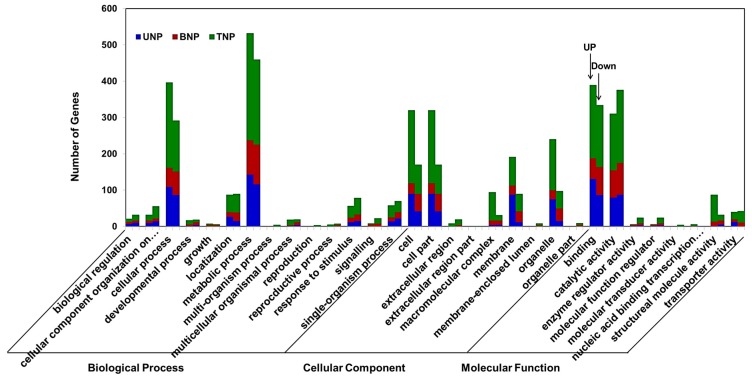
Gene Ontology (GO) classifications of differentially abundant proteins (DAPs) in KTM3315A and TM3315B during the three stages. The *x*-axis represents each GO term and the *y*-axis shows the number of enriched DAPs in each main category. “Up” represents upregulated DAPs and “down” represents downregulated DAPs. In terms of each GO term, the column on the left shows the number of upregulated DAPs and the column on the right is the number of downregulated DAPs.

GO annotation was performed using Blast2GO in the three stages in order to identify significantly enriched GO functional groups for the DAPs, as shown in Figure 4 (detailed information is provided in Table S4 and Figure S1). Compared with TM3315B, the 413 DAPs found in KTM3315A during the UNP stage were annotated according to 27 functional groups, where 12 GO terms were biological processes (the most representative were “metabolic and cellular processes” where the number of upregulated DAPs exceeded that of downregulated DAPs), eight GO terms were cellular components (the most representative were “cell and cell parts” where the number of upregulated DAPs exceeded that of downregulated DAPs) and seven GO terms were molecular functions (the most representative was “binding” where the number of upregulated DAPs exceeded that of downregulated DAPs). During the BNP stage, 373 DAPs were classified according to 28 functional groups, including 13 biological processes (the most representative were “metabolic and cellular processes” where the number of upregulated DAPs was lower than that of downregulated DAPs), eight cellular components (the most representative were “cell and cell parts” where the number of upregulated DAPs was lower that of downregulated DAPs) and seven molecular functions (the most representative were “binding” and “catalytic activity” where the number of upregulated DAPs was lower than that of downregulated DAPs). During the TNP stage, we annotated 873 DAPs according to 31 functional groups, including 14 biological processes (the most representative were “metabolic and cellular processes” where the number of upregulated DAPs exceeded that of downregulated DAPs), nine cellular components (the most representative were “cell and cell parts” where the number of upregulated DAPs exceeded that of downregulated DAPs) and eight molecular functions (the most representative were “binding” and “catalytic activity” where the number of upregulated DAPs exceeded that of downregulated DEP for “binding” whereas the opposite was found for “catalytic activity”). Based on the total DAPs during the three periods, we found that the most representative terms were metabolic process, followed by cell process and binding. Importantly, the number of upregulated DAPs for each of these three terms exceeded the number of downregulated DAPs. 

**Figure 5:**
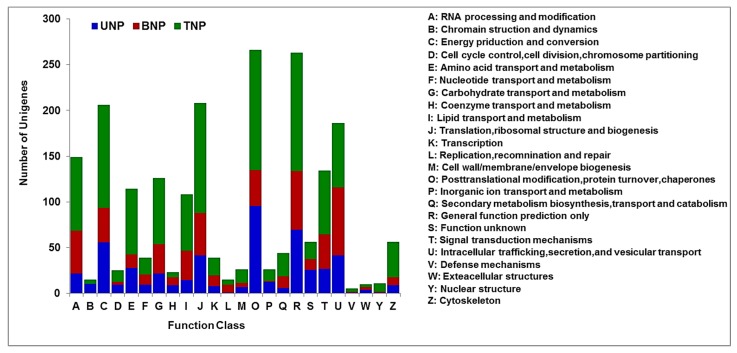
Eukaryotic Orthologous Groups of proteins (KOG) classifications for differentially abundant proteins (DAPs) in KTM3315A and TM3315B from the uninucleate stage (UNP) to the trinucleate stage (TNP). Capital letters on the *x*-axis represent the KOG categories listed to the right of the histogram. The *y*-axis shows the number of DAPs.

The DAPs identified by iTRAQ were classified according to 24 KOG categories in the UNP and TNP stages and 23 KOG categories in the BNP stage. During the UNP and TNP stages, “post-translational modification, protein turnover, chaperones” comprised the largest group (group O with 96 and 131 DAPs, respectively), followed by “general function prediction only” (group R with 70 and 128 DAPs, respectively). During the BNP stage, “intracellular trafficking, secretion and vesicular transport” (group U with 74 DAPs) was the largest group, followed by “general function prediction only” (group R with 64 DAPs) and “RNA processing and modification” (group A with 47 DAPs) (Figure 5, Table S5).

**Figure 6:**
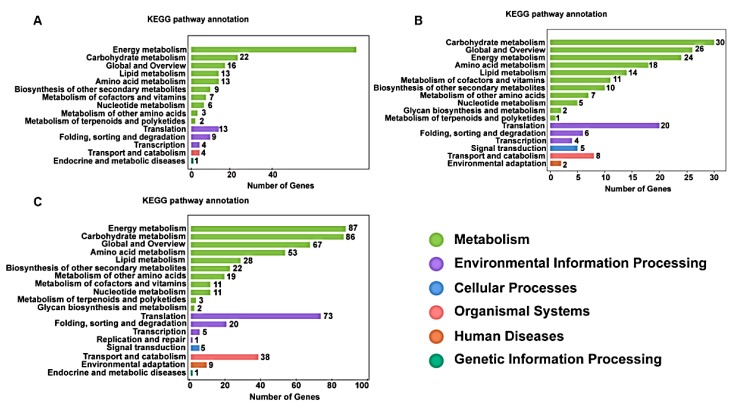
KEGG classifications of differentially abundant proteins (DAPs). The vertical axis shows the annotations of the KEGG metabolic pathways. The horizontal axis represents the gene numbers annotated in each pathway and the proportion relative to the total number of genes. (**A**–**C**) KEGG pathway classifications for DAPs in KTM3315A compared with TM3315B in the uninucleate stage (UNP), binucleate stage (BNP) and trinucleate stage (TNP), respectively. Each colored column represents a KEGG pathway and the relationships are shown on the right-hand side.

The total number of DAPs categorized as “post-translational modification, protein turnover, chaperones” was the highest among all of the KOG categories. These results suggest that post-transcriptional translation processes are important for plant growth and development. In addition, the highest number of DAPs related to the “energy production and conversion” category occurred in the TNP stage compared with the other two periods, thereby indicating that this stage is closely related to energy metabolism. The KEGG enrichment analysis for significant pathways also confirmed this finding (Figure 6).

In order to investigate the involvement of the identified proteins in biological functions in the two lines during the three stages, 204 DAPs in the UNP stage were mapped to 67 pathways in the KEGG database (Table S6), where “Photosynthesis” was the most highly represented pathway (26.96%), followed by “Oxidative phosphorylation” (12.25%) and “Starch and sucrose metabolism” (6.37%). A small number of DAPs were involved in “Cysteine and methionine, Tyrosine, Tryptophan metabolism” (0.49%) and “Benzoxazinoid biosynthesis” (0.49%). During the BNP stage, 160 DAPs mapped to 82 pathways in the KEGG database, where “Ribosome” was the most highly represented pathway (15.0%), followed by “Oxidative phosphorylation” (11.25%) and “Biosynthesis of amino acids” (8.13%). A small number of DAPs were involved in “Histidine, Tyrosine,” and Tryptophan metabolism” (0.63%) and “Benzoxazinoid biosynthesis” (0.63%). During the TNP stage, 465 (87.4%) DAPs were mapped to 90 pathways in the KEGG database, where “Ribosome” was the most highly represented pathway (18.49%), followed by “Carbon metabolism” (10.75%) and “Biosynthesis of amino acids ” (9.25%). A small number of DAPs were involved in “Glycerolipid, Tryptophan metabolism” (0.22%) and “Benzoxazinoid biosynthesis” (0.22%). The KEGG enrichment results demonstrated that most of the DAPs were involved in carbohydrate and energy metabolism pathways (Figure 6A–D), e.g., oxidative phosphorylation, TCA cycle, and starch and sucrose metabolism. For TCA cycle (also known as the citric acid cycle) pathway, there were four DEGs in the UNP stage, four in the BNP stage and 20 in the TNP stage, thereby indicating an increasing trend. DEGs involved in oxidative phosphorylation were also greatly enriched, with 25, 18 and 41 DEGs annotated in the UNP, BNP and TNP stages, respectively. Previous studies have demonstrated that TCA cycle has a key role in plant male sterility [4]. Oxidative phosphorylation provides 95% of the energy utilized by organisms and it has a significant effect on natural male sterility.

### 2.5. Analysis of DAPs in Two Important Metabolism Pathways during Pollen Development

**Figure 7:**
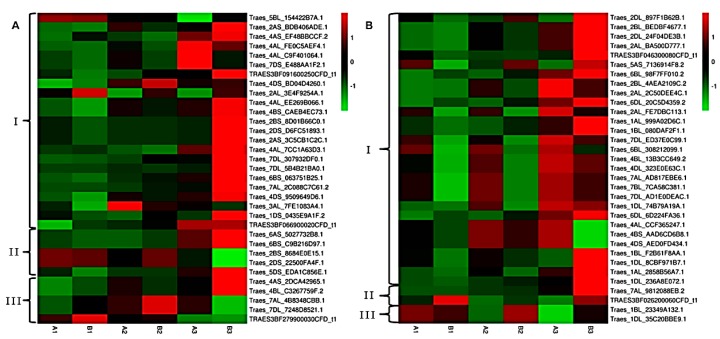
Hierarchical clustering analysis of differentially abundant proteins (DAPs) with different functions during grain development. (**A**) DAPs involved in energy metabolism, including starch and sucrose metabolism (A-I), glycolysis/gluconeogenesis (A-II) and pyruvate metabolism (A-III). (**B**) DAPs involved in carbohydrate metabolism, including oxidative phosphorylation (B-I), nitrogen metabolism (B-II) and photosynthesis (B-III).

Previous studies have demonstrated that plant male sterility is related to carbohydrate and energy metabolism. Thus, we selected 66 DAPs related to carbohydrate and energy metabolism from among the total DAPs and we aimed to determine their trends by clustering analysis. We performed hierarchical clustering analysis to determine the dynamic expression patterns of the 66 DAPs involved in carbohydrate metabolism (Figure 7A) and energy metabolism (Figure 7B). In total, 33 DAPs involved in carbohydrate metabolism were separated into three functional groups comprising starch and sucrose metabolism (A-I), glycolysis/gluconeogenesis (A-II) and pyruvate metabolism (A-III). According to Figure 7B, the 33 DAPs involved in energy metabolism were also separated into three functional groups comprising oxidative phosphorylation (B-I), nitrogen metabolism (B-II) and photosynthesis (B-III). Figure 7 shows that during the TNP stage, more genes were upregulated in the TM3315B plants compared with the KTM3315A plants. This indicates that the upregulation of more genes associated with carbohydrate and energy metabolism in TM3315B plants could ensure their normal fertility.

### 2.6. ATP and Total Sugar Assays

**Figure 8:**
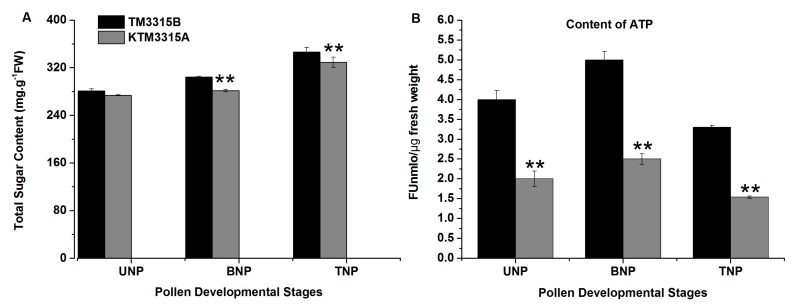
ATP and total soluble sugar contents of anthers. (**A**) Total soluble sugar contents of anthers from the maintainer line (TM3315B) and sterile line (KTM3315A) during different developmental stages. (**B**) ATP content of anthers from the uninucleate (UNP) to trinucleate stages (TNP). Data represent the mean and standard deviation based on three replicates. ** *p* < 0.01 according to the Student’s *t*-test.

In order to verify the results described above, we measured the amounts of ATP using a spectrophotometric method and the results are shown in Figure 8B, which indicates that the amount of ATP was significantly lower in the KTM3315A anthers than the TM3315B anthers from the UNP stage to the TNP stage. Thus, energy production was reduced in the KTM3315A anthers. Due to the lack of inclusions formed in the sterile pollen grains (Figure 1K) as well as the modified protein expression patterns for the carbohydrate metabolism pathways of sterile plants compared with fertile plants, we hypothesized that the sterile anthers could have been defective with respect to sugar accumulation and starch synthesis. According to the assays, we found that the total soluble sugar contents increased from the UNP stage to the TNP stage in the KTM3315A anthers (Figure 8A). There were no differences in the total soluble sugar contents in the KTM3315A anthers in the UNP stage compared with the TM3315B anthers but the contents were significantly lower in the two subsequent developmental stages (Figure 8A). In addition, the TM3315B pollen contained an abundance of starch according to its dark staining with I_2_–KI (Figure 1L). However, the KTM3315A pollen was only stained weakly by I_2_–KI (Figure 1K), which suggested more limited starch synthesis. These results indicate that the modified gene expression patterns in the carbohydrate metabolism pathway decreased the accumulation of total sugars and limited starch synthesis in the KTM3315A anthers, which might be associated with male sterility. These findings also demonstrate that the iTRAQ results were reliable.

### 2.7. Reactive Oxygen Species (ROS) Assays Using Anthers

**Figure 9:**
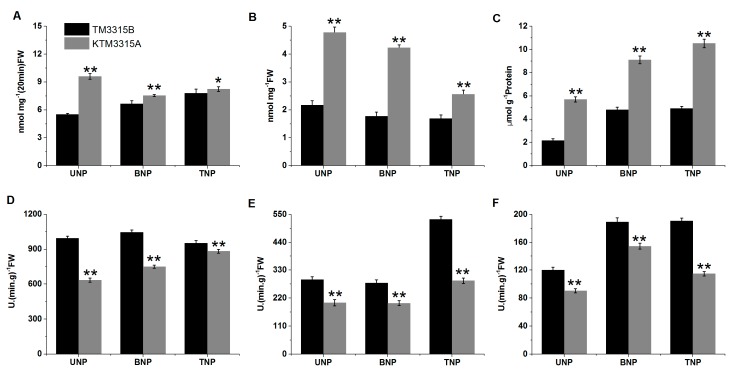
The O^2−^ production rate (**A**), H_2_O_2_ (**B**) and MDA (**C**) contents and activities of superoxide dismutase (SOD) (**D**), peroxidase (POD) (**E**) and catalase (CAT) (**F**) in developing anthers. Data represent the mean ± standard deviation based on three independent experiments. Significant differences were determined using the Student’s *t*-test (* *p* < 0.05, ** *p* < 0.01). UNP, uninucleate stage; BNP, binucleate stage; TNP, trinucleate stage.

ROS are important products related to oxidative stress in animal and plant cells, as well as being key mediators of programmed cell death (PCD) in plant and animal cells. Cells are destroyed when the ROS contents exceed a certain threshold. Thus, the ROS contents of the anthers were analyzed in order to determine whether ROS mediated the abnormalities in the KTM3315A microspores (Figure 9A–C). The results showed that the rate of ROS production was significantly higher in KTM3315A during TNP than that in the maintainer line (*p* < 0.05). In addition, the rate of ROS production during the other two stages, as well as the hydrogen peroxide (H_2_O_2_) and malondialdehyde (MDA) contents from UNP to TNP, were significantly higher than those during the same stages in the maintainer line (*p* < 0.01). These results suggest that excess ROS may contribute to male sterility in KTM3315A.

### 2.8. ROS-Scavenging Enzyme Activities

Guaiacol peroxidase (POD), superoxide dismutase (SOD) and catalase (CAT) are the major active oxygen-scavenging enzymes responsible for maintaining the normal redox status of cells and protecting them from damage. Oxidative stress is accompanied by changes in the antioxidant enzyme gene expression levels as well as their activities. In order to understand the roles of antioxidant enzymes in PCD by premature microspores in the CMS line, we measured the activities of POD, SOD and CAT in the anthers from UNP to TNP (Figure 9D–F). SOD is one of the most important active oxygen-scavenging enzymes, where it catalyzes the conversion of O^2−^ to O_2_ and H_2_O_2_. Our results showed that the SOD activity in TM3315B remained at a high level throughout the three stages of anther development. By contrast, the SOD activity remained low in the sterile line anthers, where it was significantly (*p* < 0.01) lower compared with that in the maintainer line. The POD activity was significantly lower in the sterile line during the three stages compared with the maintainer line (*p* < 0.01). CAT catalyzes the decomposition of H_2_O_2_ to produce O_2_ and H_2_O. As shown in Figure 9F, the CAT activity levels differed significantly between two lines from UNP to TNP, where it was significantly lower in the sterile line compared with the maintainer line (*p* < 0.01). Therefore, we conclude that the reduced activity of active oxygen-scavenging enzymes may prevent the rapid and effective elimination of ROS from cells, which could contribute greatly to male sterility in KTM3315A. 

### 2.9. Relationships between DAPs and Corresponding Transcripts

**Figure 10:**
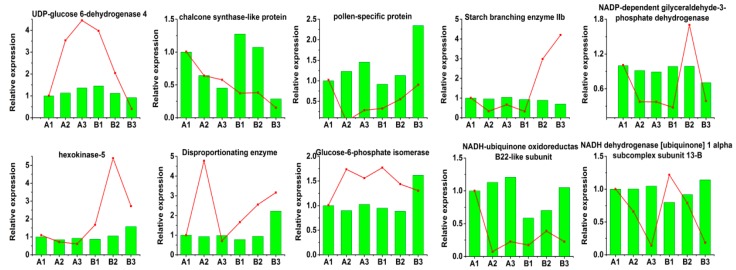
Relative expression levels of some differentially expressed proteins. The red line shows the qRT-PCR results and the histogram represent the proteomics data. The data obtained by qRT-PCR are the means based on three replicates.

In order to evaluate the correlations between the mRNA and protein levels, we employed qRT-PCR to determine the dynamic transcriptional expression patterns of 11 representative DAPs, as shown in Figure 10. We found that three genes encoding UDP-glucose 6-dehydrogenase 4, NADP-dependent NAPDH and chalcone synthase-like protein had similar expression patterns to their protein levels, while four genes encoding pollen-specific protein SF3-like, disproportionating enzyme, NADH ubiquinone oxidoreductase B22-like subunit and hexokinase (HK)-5 had similar expression patterns compared to their protein expression patterns in two or three developmental stages. However, the expression patterns of three genes encoding starch branching enzyme IIb, glucose-6-phosphate isomerase and NADH dehydrogenase 1 alpha subcomplex subunit 13-B were the opposite of their protein expression patterns. Previous studies have also indicated that the results obtained by proteomics analysis do not necessarily agree with the results of transcriptional analyses [16]. The differences between the transcriptional patterns and proteomics analyses for these four proteins might be explained by post-translation modification [17,18].

## 3. Discussion

It is well known that the development of stamens and pollen is an intensive energy-demanding process [19,20]. The most basic metabolic pathways are carbohydrate and energy metabolism, where their main physiological functions are the provision of energy and carbon sources [21]. In the present study, the iTRAQ results showed that most of the pathways included DAPs related to carbohydrate and energy metabolism, mainly the glycolysis pathway, TCA cycle and oxidative phosphorylation pathway (detailed information is provided in Table S7). If enzymes in the glycolysis pathway are inhibited, this will lead to a decrease in the amount of pyruvate as a respiratory substrate, where this change will affect the electron transport chain indirectly by influencing TCA cycle. Furthermore, previous research has shown that damage to TCA cycle can lead to male sterility [4]. However, when the electron transport chain is inhibited, excess electrons interact directly with oxygen molecules to produce ROS and this excess ROS leads to peroxidation of membrane lipids [22]. Moreover, previous research indicates that ROS are key regulators of PCD [23]. Thus, excess ROS may trigger PCD to cause male sterility. Therefore, we conducted a detailed investigation of the relationships between these three metabolic pathways and male sterility.

### 3.1. DAPs Involved in the Glycolysis Pathway

DAPs such as hexokinase (HK), 6-phosphofructokinase (PFK) and pyruvate kinase (PK) are involved in glycolysis and they were significantly downregulated in KTM3315A. These three enzymes have crucial roles in the glycolysis pathway where they all catalyze irreversible chemical reactions, i.e., HK catalyzes the first step in the glycolysis process, PFK is the catalyst of the transformation of fructose 6-phosphate into 1,6-diphosphate fructose and PK catalyzes the final step of glycolysis. In particular, PK is the catalyst of the transfer of a phosphate group from phosphoenolpyruvate to ADP to yield one molecule of pyruvate and one ATP molecule. The glycolysis pathway facilitates the conversion of glucose into pyruvate, which can be used as a respiratory substrate [24]. 

Moreover, there were differences in the expression of non-phosphorylating glyceraldehyde-3-phosphate dehydrogenases (GAPN or NP-GADPH EC 1.2.1.9) in KTM3315A and TM3315B. NP-GADPH is the catalyst of the irreversible NADPH-dependent oxidation of GAP into 3-phosphoglycerate and NADPH [25]. This reaction is required for the glycolytic “bypass” pathway found uniquely in photosynthetic eukaryotes, including both plants and microalgae, which circumvents the first substrate level phosphorylation step of glycolysis [26]. This reaction is the major source of the NADPH utilized for mannitol biosynthesis in many plant species [27]. Pyruvate dehydrogenase is a key regulator in TCA cycle and a previous study showed that inhibiting the activity of pyruvate dehydrogenase in the anthers was sufficient to cause male sterility in sugar beet [28]. According to the present study, the iTRAQ results indicated that the pyruvate dehydrogenase E2 component was downregulated in KTM3315A compared with TM3315B, which may be important for the mechanism of male sterility (CMS). 

In addition, other DAPs related to the glycolysis pathway such as glucose-6-phosphate isomerase, fructose-bisphosphate aldolase and enolase were all downregulated during the TNP stage in KTM3315A. Therefore, the amount of pyruvate that entered TCA cycle was reduced and thus the amount of respiratory substrate decreased, thereby affecting the electron transport chain indirectly. This may have an impact on the development of anthers. 

### 3.2. DAPs Involved in TCA Cycle

Previously, it was demonstrated that defects in TCA cycle may cause male sterility [3]. In the present study, 13 DAPs were involved in TCA cycle during TNP in KTM3315A, where 10 DAPs were downregulated and the only three were upregulated, such as ATP-citrate lyase, which catalyzes the conversion of citrate and CoA into acetyl-CoA and oxaloacetate, as well as ATP hydrolysis. The 10 downregulated enzymes included two citric acid synthases and three IDHs, which are critical enzymes in TCA cycle. Citric acid synthases catalyze the conversion of citric acid and CoA to generate acetyl-CoA and oxaloacetic acid in the citrate cycle. IDH catalyzes the oxidative decarboxylation of isocitrate to produce alpha-ketoglutarate (α-ketoglutarate) and CO_2_, where this two-step process comprises the oxidation of isocitrate (a secondary alcohol) to oxalosuccinate (a ketone), which is followed by decarboxylation of the carboxyl group beta to the ketone to form alpha-ketoglutarate. This reaction is irreversible and it is the rate-limiting step in TCA cycle. Other enzymes downregulated in TCA cycle included three succinyl-CoA synthetases and two fumarate hydratases. These results clearly suggest that all of these changes may have reduced the amounts of coenzymes (NADH and FADH_2_) produced in TCA cycle, so less coenzyme entered the respiratory chain and the formation of ATP was decreased (Figure 8B). A previous study showed that the key enzymes decreased in the glycolysis and TCA cycles, which also led to decreases in the ATP content [3]. Moreover, many studies have detected lower ATP production in some CMS flowers [29,30,31]. According to these results, we suggest that the energy demand is not met for a short period during the plant development process, thereby causing pollen abortion in KTM3315A.

### 3.3. DAPs Disturbed the Electron Transport Chain and ATP Synthesis

In this study, compared with TM3315B, DAPs comprising complex I (NADH dehydrogenase), complex IV (cytochrome oxidase), complex III (cytochrome reductase) and complex V (ATP synthase) were all downregulated in the electron transport chain during TNP in KTM3315A. NADH dehydrogenase is the first enzyme in the mitochondrial electron transfer chain. Initially, NADH binds to complex I before transferring high energy electrons to subsequent complexes (complex III, complex IV and complex V). Complex I is a major site of premature electron leakage to oxygen [32] and it also has a role in triggering apoptosis [33,34]. Previous studies have also shown that when electron transfer is reduced, the excess electrons combine directly with molecular oxygen to produce excessive amounts of ROS [22]. In this study, the downregulation of those proteins in the electron transport chain resulted in the electron transfer chain being blocked to promote ROS production (Figure 9A–C). However, if the ROS cannot be eliminated rapidly, cells will be affected by oxidative stress, which will cause intracellular damage and even trigger cell death [35,36]. Oxidative stress leads to increases in mitochondrial electron transport, as well as enhancing H_2_O_2_ production and depleting ATP [37], as also shown in our study (Figure 8B and Figure 9A–C). In addition, a series of antioxidant enzymes were downregulated according to our iTRAQ results during the TNP stage in KTM3315A wheat, as found in previous studies [34,38]. The activities of active oxygen-scavenging enzymes in the three stages were significantly lower in the sterile lines compared with the maintainer lines (Figure 9D–F). These reductions in antioxidant and active oxygen-scavenging enzymes indicate that the formation of sterile microspores in KTM3315A is related to oxidative stress. 

In addition, excessive amounts of ROS can lead to changes in mitochondrial permeability [39]. It is generally considered that the generation of excessive amounts of ROS leads to apoptotis and necrotic cell death [40,41]. Moreover, three cytochrome reductase subunits and two cytochrome oxidase subunits were downregulated at TNP in KTM3315A. Structural changes in cytochrome reductase will clearly affect its function, thereby affecting electron transport from coenzyme Q to oxygen. Changes in cytochrome oxidase will also affect the electron transport from cytochrome C to oxygen. Therefore, the downregulation of these proteins would inhibit the electron transport chain, thereby affecting pollen development. 

Furthermore, we identified four subunits of ATP synthase as DAPs. Mitochondrial membrane ATP synthase (F1F0 ATP synthase or complex V) has an important role in energy metabolism where it converts ADP into ATP in the presence of a transmembrane proton gradient [42]. Recent analyses of plant mitochondrial complexes have demonstrated that several mitochondrial DNA regions that encode these ATP synthase subunits are related to male sterility [42,43,44]. In the present study, we found that ATP synthase precursor subunit ε and subunit d were downregulated, which might have made ATP synthase dysfunctional and affected the mitochondrial energy output to induce changes in the mitochondrial membrane potential, thereby leading to abnormal anther development with nonfunctional pollen. Thus, our results suggest that the downregulation of these proteins slowed down the electron transport rate in KTM3315A plants but excess electrons were generated and they combined with molecular oxygen to form ROS in pollen development, thereby initiating PCD in the anthers. Recently, many studies of plant male sterility have focused increasingly on ROS metabolism [45,46]. The aberrant expression of mitochondrial genes may affect the greater demand for the respiratory function and cellular energy in the form of ATP during the development of anthers [22]. Our analyses of the ATP contents also support this viewpoint (Figure 8B), thereby giving novel insights into the molecular mechanisms responsible for male sterility in wheat. 

### 3.4. A Possible Protein Regulation Network Related to Male Sterility in Wheat

**Figure 11:**
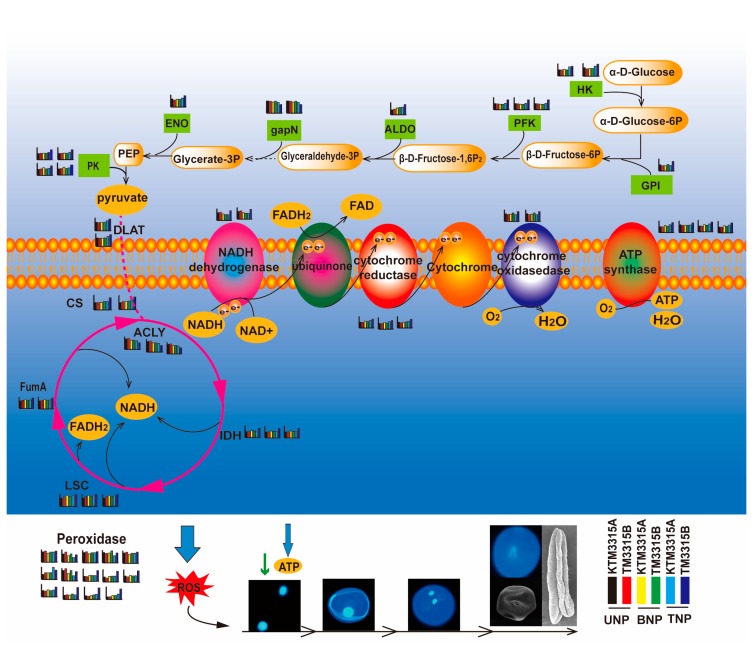
Possible protein network responsible for controlling male sterility in wheat. Histograms and numbers represent the protein identifications and abundance levels given in Supplementary Table S7. CS, citrate synthase; ACLY, ATP citrate (pro-S)-lyase; IDH, isocitrate dehydrogenase; FumA, fumarate hydratase; LSC, succinyl-CoA synthetase alpha subunit; DLAT, pyruvate dehydrogenase E2 component; HK, hexokinase; GPI, glucose-6-phosphate isomerase; PFK, 6-phosphofructokinase 1; ALDO, fructose-bisphosphate aldolase; GAPN, glyceraldehyde-3-phosphate dehydrogenase (NADP+); ENO, enolase; PK, pyruvate kinase; UNP, uninucleate stage; BNP, binucleate stage; TNP, trinucleate stage.

According to the KEGG cluster analysis of DAPs, we analyzed the DAPs in the three metabolic pathways (glycolysis, TCA cycle and pyruvate metabolism) related to carbohydrate and energy metabolism, as well as considering previous studies [22] and we propose a protein regulatory network that may be responsible male sterility in KTM3315A. As shown in Figure 11, the downregulation of enzymes related to glycolytic metabolism ultimately results in a decrease in the amount of pyruvate in the citric acid cycle, which affects the citrate cycle to some extent. In addition, downregulation of the DAPs in the citric acid cycle decreases the number of coenzymes (FADH_2_ and NADH), thereby reducing the coenzymes entering the electron transport chain. Defects in TCA cycle are known to be sufficient to cause male sterility [3]. In the electron transport chain, electron transfer is inhibited due to the downregulation of the key complexes and antioxidant enzymes, so the electrons combine directly with molecular oxygen to generate excessive amounts of ROS [22], where the reduced activity of active oxygen-scavenging enzymes and the downregulation of antioxidants means that ROS cannot be eliminated sufficiently rapidly and thus cells undergo oxygen stress. Oxygen stress promotes the production of H_2_O_2_ (Figure 9B) and the consumption of ATP [37] (Figure 8B). Moreover, the abnormal expression of ATP synthase affects the mitochondrial energy output and induces changes in the mitochondrial membrane potential to cause abnormal anther development and ultimately pollen abortion.

### 3.5. Differences between the Proteome Data and qRT-PCR Results

The development of high-throughput techniques for identifying the expression patterns of thousands of genes has greatly enhanced our understanding of biological processes. However, the occurrence of post-translational modifications and other biological processes suggests that mRNA is not a reliable indicator of protein levels. Moreover, previous studies indicate that the correlations are poor between the mRNA and protein levels for all except the most abundant proteins [47,48]. In addition, the activity of a protein depends on many factors such as its location and half-life and a protein might need to be modified or interact with other proteins before it becomes active. Indeed, the inherent complexity of proteins means that there is no clear consistent relationship between the mRNA and protein expression levels. Thus, elucidating the correlations between protein and mRNA expression levels also requires some consideration of possible post-translational modifications.

## 4. Materials and Methods

### 4.1. Plant Materials

KTM3315A is an *Aegilops kotschyi* CMS line that carries chromosome 1BS from *Triticum macha*, which exhibits complete male sterility during the normal season and its fertility restoration is better than that of the 1B/1R wheat CMS lines with *Ae. kotschyi* cytoplasm. The wheat CMS line was developed via consecutive backcrosses with TM3315B as the donor parent [9]. The plant materials used in this study were grown in an experimental field at Northwest A&F University, Yangling, China. We collected several spikes in order to identify the developmental stages, where we observed the pollen mother cells during meiosis and the subsequent microspore development with conventional smear squashing and acetic red dyeing methods. We collected the anthers from the middle florets of the main spikes of KTM3315A and TM3315B during the UNP, BNP and TNP stages of pollen development. The harvested anthers were immediately placed in liquid nitrogen, before storing at −80 °C until they were used in experiments.

### 4.2. Phenotypic Characterization and Cytological Observations

Photograph images of the plant materials were acquired using a Nikon E995 digital camera (Nikon, Tokyo, Japan) on a Motic K400 dissecting microscope (Preiser Scientific, Louisville, KY, USA). The different stages of anther development were identified by staining with 1% acetocarmine. The chromosomes were analyzed after staining with DAPI and producing paraffin sections, which were prepared as described by Sheng [49]. Dehiscent anthers from mature flowers were stained with I_2_–KI (1 g iodine and 3 g potassium iodide in 100 mL of water) [50] to evaluate the viability of the mature pollen grains. Anthers and microspores were subjected to SEM with a JSM-6360LV scanning electron microscope (JEOL, Tokyo, Japan) according to the method of Zhang et al. [51].

### 4.3. Protein Extraction and Quantification

Proteins were extracted from anthers using the method employed by Li et al. [4] with some modifications. Samples of the wheat anther were ground in liquid nitrogen to obtain a powder and extracted with Lysis Buffer 3 (8 M Urea, 2 M thiourea, 4% CHAPS, 40 mM Tris–HCl, pH 8.5), which contained 2 mM EDTA and 1 mM phenylmethane sulfonyl fluoride (PMSF). Next, 10 mM dithiothreitol (DTT) was added after 5 min, before sonicating for 5 min and subjecting to centrifugation at 4 °C and 25,000× *g* for 20 min. The supernatant obtained was mixed with five volumes of chilled acetone containing 10% (*v*/*v*) trichloroacetic acid, before adding 10 mM DTT and incubating at −20 °C for 2 h. Centrifugation was performed at 4 °C and 25,000× *g* for 20 min and the supernatant was discarded. Next, 1 mL of cold acetone containing 10 mM DTT was added to the precipitate, before centrifugation at 4 °C and 25,000*×* *g* for 20 min and this process was repeated twice. After drying in the air, the precipitate was dissolved in Lysis Buffer 3, which contained 1 mM PMSF and 2 mM EDTA. Next, 10 mM DTT was added after 5 min and the suspension was sonicated for 5 min, before centrifugation at 4 °C and 25,000× *g* for 20 min. Subsequently, 10 mM DTT was added to the supernatant, before incubating for 1 h at 56 °C and then alkylating with 55 mmol/L iodoacetamide for 45 min in the dark at room temperature. The suspension was diluted five times with 80% trichloroacetic acid and incubated for 2 h at −20 °C, before centrifuging at 25,000× *g* and 4 °C for 20 min. The supernatant was discarded. The precipitate was air dried, before redissolving in Lysis Buffer 3, sonicating for 5 min and centrifuging for 20 min at 4 °C and 25,000× *g*. The supernatant was either stored at −80 °C or analyzed immediately. The protein concentration was analyzed using the Bradford assay with several different concentrations of bovine serum albumin as the standard [52]. Sodium dodecyl sulfate-polyacrylamide gel electrophoresis was employed to confirm the protein quality and concentration. Proteins in the supernatant were stored at −80 °C until subsequent analyses. 

### 4.4. iTRAQ Analysis

iTRAQ analysis was conducted by Guangzhou Genedenovo Biotechnology Co., Ltd. (Guangzhou, China). Protein samples (100 μg each) from the anthers of the two lines were digested in Trypsin Gold (Promega, Madison, WI, USA) for 16 h at 37 °C. The peptide segment in each sample was labeled using an iTRAQ 8-plex kit (Applied Biosystems, Foster City, CA, USA) according to the manufacturer’s instructions. Maintainer line samples from the UNP, BNP and TNP stages were labeled with 113,114 and 115 tags, respectively, whereas the sterile line UNP, BNP and TNP samples were labeled with 116, 117 and 118 tags. Different tags were separated according to the ion mass following neutral loss of the balance group. The samples were pooled after labeling and resuspended in strong cation exchange (SCX) loading buffer before fractionation using SCX chromatography on an Ultremex SCX column (4.6 × 250 mm) with a 3000 high-performance liquid chromatography system (Thermo Fisher DINOEX, Waltham, MA, USA). Data were acquired by a TripleTOF 5600+ System (AB SCIEX, Framingham, MA, USA) fitted with a Nanospray III source and a pulled quartz tip as the emitter (New Objectives, Woburn, MA, USA). Proteins were identified and quantified using Mascot software (version 2.3.02, Matrix Science Inc., Boston, MA, USA). The search parameters employed were set as follows: peptide tolerance = 20 ppm and fragment mass tolerance = 0.05 Da; tryptic peptides with ≤1 missed cleavage site; threshold set-off = 0.05 in the ion-score cut-off; variable oxidation of methionine, pyrophosphorylation of glutamine and iTRAQ labeling of tyrosine were set as variable modifications; and iTRAQ labeling of lysine, carbamido methylation of cysteine and N-terminal amino group of peptides were set as fixed modifications. iTRAQ 8-plex was employed for simultaneous quantification in the search process. Before exporting the data, the search results were subjected to additional filters, which were set as follows for protein identification: significance threshold *p* < 0.05 (95% confidence) and ion score or expected cutoff <0.05 (95% confidence). For protein quantification, the filters were set as follows: minimum precursor charge = 2, “median” for protein ratio type and unique spectrum = 2, with normalization by median intensities and any outliers were removed automatically. The peptide threshold was set as defined above for identification. Searches were performed against the coding sequence (CDS) protein database for wheat (Ensembl version 30, 100,344 proteins). Changes of 1.2-fold or <0.83-fold were used to identify significant DAPs together with a *p*-value < 0.05. More details of the iTRAQ method are given in the Supporting Information S1.

### 4.5. Bioinformatics Analysis

The DAPs identified in KTM3315A and TM3315B were functionally annotated using Blast2GO and the non-redundant protein database (NR; NCBI: http://www.geneontology.org/). Functional classification was performed based on the Eukaryotic Orthologous Groups of proteins (Available online: http://www.ncbi.nlm.nih.gov/COG/) for all the DAPs using Blastx 2.2.29+ with the STRING database (Available online: http://string-db.org/, version 10.5). Subsequently, each DEP was mapped to a pathway in the KEGG database (Available online: http://www.genome.jp/kegg/genes.html) with BLASTx/BLASTp 2.2.29+. *P* ≤ 0.05 was used to confirm the significance of the GO and KEGG pathway analysis results. OmicShare small tools^2^ was employed to obtain a heatmap with threshold parameters of no rows and column clusters. 

### 4.6. Quantification of Reactive Oxygen Species 

The contents of O_2_^−^, hydrogen peroxide, malondialdehyde, as well as the catalase, superoxide dismutase and guaiacol peroxidase activity levels were determined according to the method of Ba et al. [53].

### 4.7. Total Sugar Assay

The anthers were collected and frozen at −80 °C, before grinding to a fine powder in 2 mL of 80% ethanol solution using a mortar and pestle, incubating at 80 °C for 40 min and then centrifuging at 3000× *g* for 15 min. The supernatant was placed in another tube and 2 mL of 80% ethanol solution was added to the residue before repeating the first step. The supernatants were then combined in the same test tube and the volume was made up to 5 mL by adding 80% ethanol solution. Next, 1 mL of a solution containing fructose, glucose, or galactose was prepared. The standard used for optimization was a 200 μg/mL glucose solution. The total sugar contents were determined for the fertile and male sterile anthers with the anthrone colorimetric method [54].

### 4.8. ATP Assay

The spectrophotometric method used to determine the ATP contents followed the protocol provided with an ATP Detection Kit (Comin Biotechnology Co., Ltd., Suzhou, China). The wheat anthers were collected and ground immediately on ice with 1 mL of acid extract from the ATP Detection Kit. Centrifugation was performed for 10 min at 4 °C and 8000× *g* and the supernatant was transferred to a new 1.5 mL tube, before then adding the same volume of alkaline extract and mixing and centrifuging for 10 min at 4 °C and 8000× *g*. The supernatant was placed in a new tube on ice for the ATP assay. The luminescence was assayed from a 30-μL sample using a spectrophotometer with 100 μL of the ATP detection buffer provided in the ATP Detection Kit. All of the experiments were repeated in triplicate and the mean values were determined based on the three replicates (±standard deviation).

### 4.9. Quantitative Real-Time PCR (qRT-PCR)

Sequence-specific primers were designed with Primer-BLAST (Available online: https://www.ncbi.nlm.nih.gov/tools/primer-blast/) and synthesized by Sangon Biotech (Shanghai) Co., Ltd., China. The actin gene (GenBank: GQ339766.1) was used as a reference for normalizing the gene expression levels, where it was set at 1. The qRT-PCR analysis was performed as described by Ye et al. [55]. The primer pairs used for qRT-PCR are given in Table S8. The analysis was conducted using at least three replicates. We calculated the relative gene expression levels using the 2^−∆∆*C*t^ method [56]. 

## 5. Conclusions

According to cytological studies, we found structural differences in the anthers and pollen grains from a sterile line and maintainer line in wheat. However, the mechanism of sterility is still unclear. In order to obtain deeper insights into the mechanism responsible for male sterility, we used iTRAQ to identify DAPs in the anthers of the sterile line and maintainer line in wheat, before conducting cluster analysis based on the DAPs to gain insights into male sterility. KEGG clustering analysis of the DAPs showed that carbohydrate and energy metabolism were closely related to male sterility and the hierarchical clustering results showed that more DAPs associated with energy and carbohydrate metabolism were upregulated in the maintainer line compared with the sterile line. Thus, the upregulation of certain key proteins is a prerequisite for ensuring the fertility of wheat. Based on analyses of these metabolic pathways and previous research, we proposed a possible protein regulatory network that determines the fertility of wheat. We consider that the downregulation of key proteins in this regulatory network means that pollen grains are affected by oxidative stress, which is the main cause of male sterility in KTM3315A wheat. Furthermore, this regulatory network was verified by determining the activities of ATP, ROS and ROS-scavenging enzymes. We conclude that CMS in wheat is due to the effects of multiple genes rather than a single gene. Thus, our findings give new insights into the mechanisms responsible for male sterility and they will promote further studies of hybrid wheat.

## Supplementary Materials

Supplementary materials can be found at http://www.mdpi.com/1422-0067/19/2/324/s1.
