# Supplementary material for: Identification of Proteins Involved in Carbohydrate Metabolism and Energy Metabolism Pathways and Their Regulation of Cytoplasmic Male Sterility in Wheat
Source: Int J Mol Sci. 2018 Jan 23;19(2):324. doi: 10.3390/ijms19020324 (PMC5855548; doi:10.3390/ijms19020324)
Supplement: Supplementary file 1 [file ijms-19-00324-s001.zip › Suplementary Materials/Suplementary Materials-for proofreading.docx]

**Supporting Information S1: Detailed iTRAQ analysis procedure**

Protein samples (100 μg each) from the anthers of the two lines were digested with 50 mM trypsin. The peptide segment in each sample was labeled using iTRAQ 8-plex kits (AB Sciex Inc., USA) according to the manufacturer’s instructions. Maintainer line samples from the UNP, BNP and TNP stages were labeled with 113,114 and 115 tags, respectively and the sterile line samples from the UNP, BNP and TNP were labeled with 116, 117 and 118 tags respectively. The sample fractionation was performed before LC-MS/MS analysis using SCX chromatography on an Ultimate 3000 HPLC system (Thermo Fisher DINOEX, Waltham, MA, USA). The iTRAQ labeled peptide mixtures were reconstituted with 100 μL buffer A [25 mM NaH2PO4 in 25% acetonitrile (ACN), pH 2.7] and loaded onto a 4.6×250 mm Ultremex SCX column containing 5-μm particles (Phenomenex). The peptides were eluted at a flow rate of 1mL/min with a gradient of buffer A for 10 min, 5-60% buffer B (25mM NaH2PO4, 1 M KCl in 25% ACN, pH 2.7) for 27 min, 60-100% buffer B for 1 min. The system was then maintained at 100% buffer B for 1 min before equilibrating with buffer A for 10 min prior to the next injection. Elution was monitored by measuring the absorbance at 214 nm and fractions were collected every 1 min. The eluted peptides were pooled into 12 fractions, desalted with a ZipTip C18 column (Waters) and vacuum-dried. Each fraction was resuspended in buffer A [5% ACN, 0.1% formic acid (FA)] and centrifuged at 12,000g for 10 min, the final concentration of peptide was about 0.5 μg/μl on average. Ten microliter supernatants were loaded on a Eksigent nanoLC System (AB SCIEX, Framingham, MA, USA) by the autosampler onto a C18 trap column (5 μm, 100 μm×20 mm). The peptides were eluted onto a self-packed analytical C18 column (3 μm, 75 µm×150 mm). The samples were loaded at 5 μL/min for 10 min, then the 78 min gradient was run at 300 nL/min starting from 2 to 35% B (95%ACN, 0.1%FA), followed by 5 min linear gradient to 60%, then, followed by 2 min linear gradient to 80% and maintenance at 80% B for 4 min and finally return to 5% in 1 min.

Data acquisition was performed with a TripleTOF 5600+ System (AB SCIEX, Framingham, MA, USA) fitted with a Nanospray III source and a pulled quartz tip as the emitter (New Objectives, Woburn, MA). Data was acquired using an ion spray voltage of 2.5 kV, curtain gas of 35 psi, nebulizer gas of 10 psi and an interface heater temperature of 150 °C. The MS was operated in sensitive mode for TOF MS scans. For information dependent acquisition (IDA), survey scans were acquired in 250 ms and as many as 30 product ion scans were collected if exceeding a threshold of 120 counts per second (cps) and with a 2+ to 5+ charge-state. Total cycle time was fixed to 3.3 s. Q2 transmission window was 100Da for 100%. Four time bins were summed for each scan at a pulser frequency value of 11 kHz through monitoring of the 40 GHz multichannel TDC detector with four-anode channel detect ion. A sweeping collision energy setting of 35±5 eV coupled with iTRAQ adjust rolling collision energy was applied to all precursor ions for collision-induced dissociation. Precursor ions were excluded from reselection for 15 s (1/2 of average peak width). Raw data files acquired from the TripleTof were converted into MGF files using Proteome Discoverer 1.2 and the MGF files were searched. Proteins were identified and quantified using Mascot software (version 2.3.02, Matrix Science Inc., Boston, MA). The search parameters were as follows: peptide tolerance =10 ppm and fragment mass tolerance = 0.05 Da; threshold set-off = 0.05 in the ion-score cut-off; tryptic peptides with ≤ 1 missed cleavage site; pyrophosphorylation of glutamine, variable oxidation of methionine and iTRAQ labeling of tyrosine were set as variable modifications; and carbamido methylation of cysteine, iTRAQ labeling of lysine and N-terminal amino group of peptides were set as fixed modifications. iTRAQ 8-plex was employed for simultaneous quantification during the search process. The search results were passed through additional filters before exporting the data. The filters were set as follows for protein identification: significance threshold P < 0.05 (with 95% confidence) and ion score or expected cutoff < 0.05 (with 95% confidence). The filters were set as follows for protein quantification: “median” for protein ratio type, minimum precursor charge = 2 and unique spectrum = 2, with normalization by median intensities and outliers were removed automatically. The peptide threshold was set as described above for identification. Searches were performed against the coding sequence (CDS) protein database for wheat (Ensembl version 30, 100344 proteins). Changes of 1.2-fold or < 0.83-fold were used to identify significant DEPs together with a P-value < 0.05.

**Figure S1-S2**


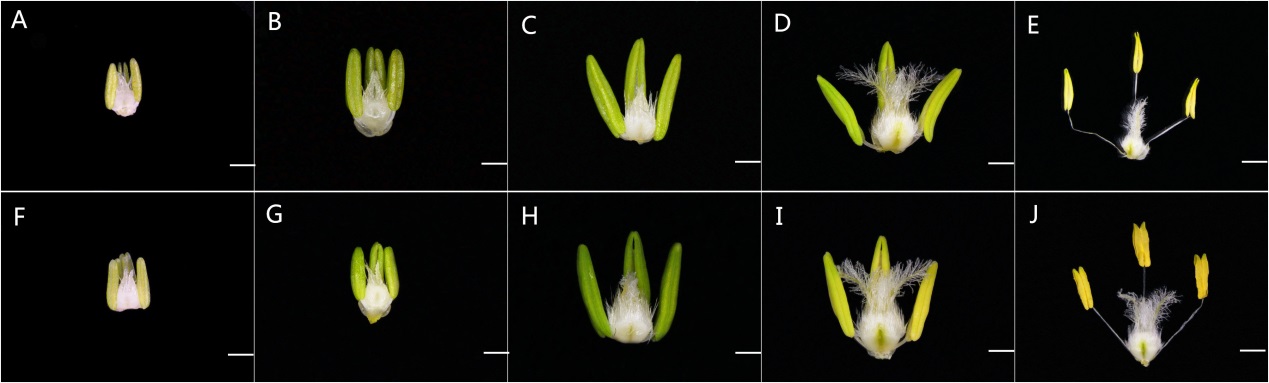


**Figure S1.** Comparison of the stamens and pistils in sterile line (SL) (A to E) and fertile line (FL) wheat plants (F to J). A and F, tetrad stage; B and G, early UNP; C and H, later UNP; D and I, BNP; E and J, TNP. Scale bars are 0.5 mm in A to J.


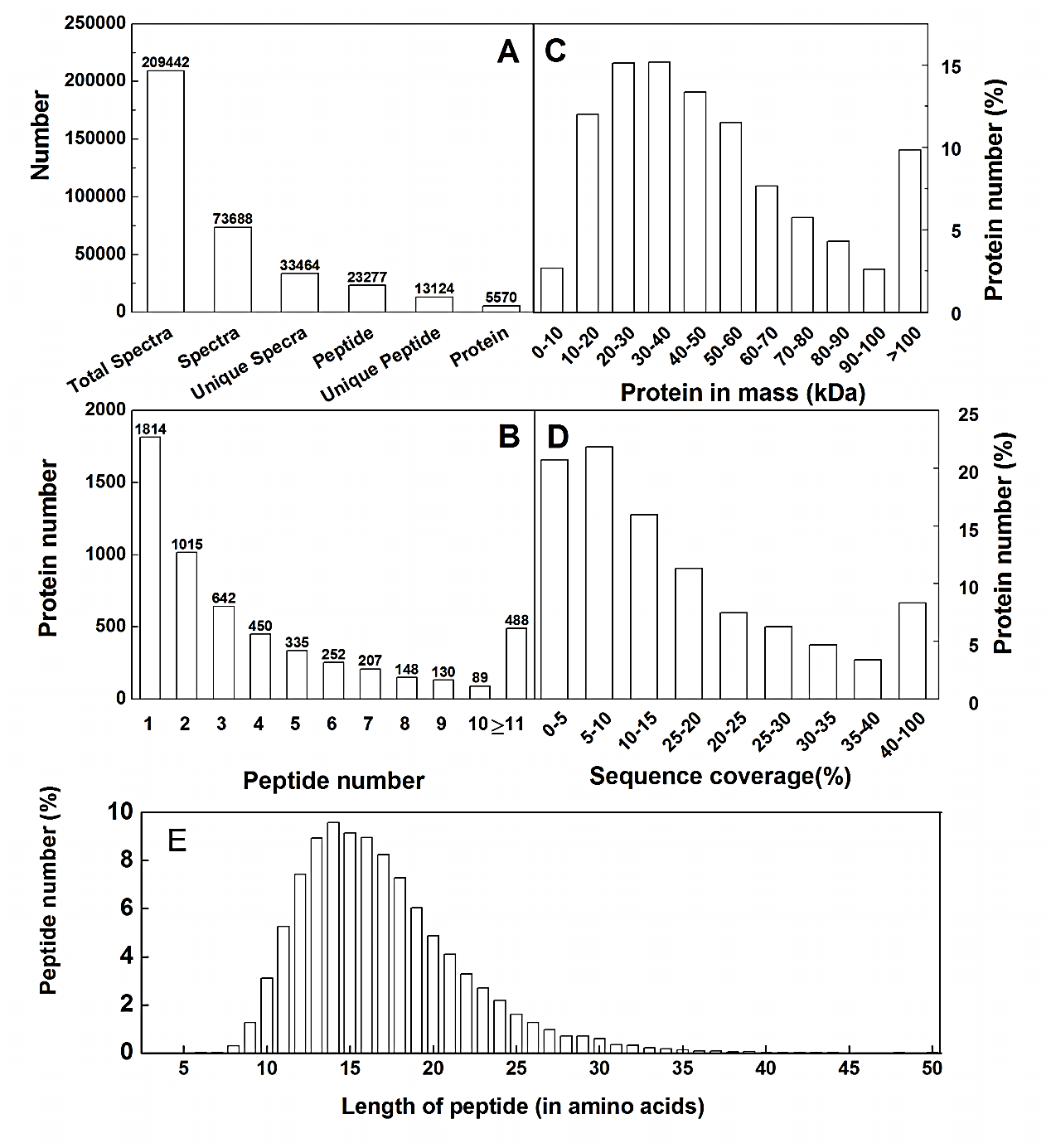


**Figure S2.** Spectra, peptides and proteins identified by iTRAQ proteomics after searching against the coding sequence (CDS) protein database for wheat (A), number of peptides that matched with proteins using MASCOT (B), protein mass distribution (C), distribution of protein sequence coverage (D) and distribution of peptide lengths (E).
